# Overexpression of an alfalfa glutathione S-transferase gene improved the saline-alkali tolerance of transgenic tobacco

**DOI:** 10.1242/bio.043505

**Published:** 2019-08-30

**Authors:** Binghao Du, Weidi Zhao, Yimin An, Yakun Li, Xue Zhang, Lili Song, Changhong Guo

**Affiliations:** Key Laboratory of Molecular and Cytogenetics, College of Life Science and Technology, Harbin Normal University, Harbin 150025, Heilongjiang Province, China

**Keywords:** Alfalfa, *MsGSTU8*, Saline-alkali tolerance, ROS, Antioxidant enzyme, Transgenic tobacco

## Abstract

Abiotic stresses restrict the productivity and quality of agricultural crops. Glutathione S-transferase (GST) utilizes glutathione to scavenge reactive oxygen species (ROS) that result from abiotic stresses. This study aimed to determine the expression pattern of the *MsGSTU8* gene and its effects on saline-alkali tolerance. *MsGSTU8*, from alfalfa (*Medicago sativa* ‘Zhaodong’), was transformed into transgenic tobacco (*Nicotiana tabacum*) and overexpressed to determine its effects on saline-alkali tolerance. The gene products in alfalfa localized to the cytoplasm and the transcript levels were higher in the leaves than the roots and stems. Expression was strongly induced by cold, drought, salt and saline-alkali stresses as well as abscisic acid (ABA) treatments. The transgenic tobacco lines had significantly higher transcription levels of the abiotic stress-related genes and higher GST activity than the wild types. Transgenic tobacco lines with saline-alkali treatments maintained their chlorophyll content, showed improved antioxidant enzyme activity and soluble sugar levels, reduced ion leakage, O_2_^.−^, H_2_O_2_ accumulation and malondialdehyde content. Our results indicate that overexpression of *MsGSTU8* could improve resistance to saline-alkali stresses by decreasing the accumulation of ROS and increasing the levels of antioxidant enzymes. Furthermore, they suggest that *MsGSTU8* could be utilized for transgenic crop plant breeding.

## Abstract (precis)

**Summary:** The glutathione S-transferase gene of *Medicago sativa* can effectively alleviate osmotic and oxidative damage induced by saline-alkali stress in transgenic tobacco.

## INTRODUCTION

Plants regularly have to cope with abiotic stresses such as cold, heat, drought, salt and saline-alkali environments during their growth and development (Yu et al., 2018), which can seriously affect their yield and quality (Bechtold and Field, 2018; Shameer and Prasad, 2018). In the process of plant evolution, plants have developed various response mechanisms, especially molecular reaction pathways and regulatory networks, in order to adapt to abiotic stresses (Qu et al., 2013). Numerous induced genes have been identified in plants under abiotic stress, including heat shock proteins (Gruber et al., 2018; Traewachiwiphak et al., 2018; Zhao et al., 2018), transcription factors (Erpen et al., 2018; Klay et al., 2018; Moreno et al., 2018), protein kinases (Manuka et al., 2018; Ye et al., 2017; Zhou et al., 2018), protein phosphatases (Singh et al., 2010; Kong et al., 2018) and cellular protective enzymes (Hanaka et al., 2018; Suzuki et al., 2013). Glutathione S-transferases (GSTs; EC 2.5.1.18) are a group of multifunctional protective cellular enzymes found in all cellular organisms and are encoded by a large complex superfamily in plants (Abdul et al., 2018; Liu et al., 2017). GSTs affect the growth and development of plants through their involvement in plant primary metabolism, secondary metabolism, stress tolerance (Horváth et al., 2019; Kumar and Trivedi, 2018; Yang et al., 2019) and cell signal transduction. GSTs catalyze the nucleophilic addition of reduced glutathione hydrosulfuryl and lipophilic electrophilic substrates, including organic halides, epoxides, arene oxides, α-unsaturated carbonyls, β-unsaturated carbonyls, organic nitrate esters and organic thiocyanates. Solubility was increased by the conjugation of glutathione to such molecules, producing water-soluble products that facilitated further metabolic processing (Axarli et al., 2009). External toxins and endogenous toxic metabolites are isolated in vacuoles or transferred to the apoplast for degradation of conjugated substances by covalent bonding, reducing the toxicity of substrates (Edwards and Dixon, 2005; Flury et al., 1995; Marrs, 1996; Moons, 2003; Sandermann, 1992). GSTs act as binding proteins or ligands and can also function as non-enzyme carriers in intracellular transport and catalyze anthocyanin-glutathione binding following transport to the vacuole through a glutathione pump (Marrs, 1996). GST can scavenge reactive oxygen species (ROS) including superoxide anions, hydroxyl radicals, alkoxys and hydrogen peroxide to protect the organism from oxidative damage (Cummins et al., 1999). GSTs also play a vital role in the isoversion reaction, redox steady-state of cells and regulation of cellular program senescence (Moons, 2005). Based on sequence similarity, immunological reactivity, kinetic properties and structural conformation, GSTs have been found to be widespread among plants.

In previous research, GST genes have been shown to be expressed in response to various abiotic stresses. For example, ROS content was reduced in transgenic tobacco overexpressing *JrGSTU1* under cold stress (Yang et al., 2016), *AtGSTU19* facilitated the maintenance of the ROS balance of cells of transgenic *Arabidopsis* by improving the activity of GST and other antioxidant enzymes that enhanced resistance to salt stress (Xu et al., 2015a), the transcriptional levels of *MaGSTU2* and *MaGSTU3* increased under drought stress (Wang et al., 2013), and overexpression of *LeGSTU2* conferred tolerance to drought stress in transgenic *Arabidopsis* (Xu et al., 2015b). Members of the GST family have been reported in many different plants, but MsGSTs that resist saline-alkali stress have yet to be investigated. Exploration of the function of *MsGSTU8* in response to saline-alkali stress would thus be of value to the scientific community.

Alfalfa (*Medicago sativa* L.), is a perennial high-quality forage legume that has received attention due to the positive impact its cultivation has on soil fertility (Gu et al., 2018; Seppänen et al., 2018). *Medicago sativa* ‘Zhaodong’, a cultivar local to northeast China, is tolerant to saline-alkali stress, and can grow on saline-alkali soils without negative effects. The transcriptome of alfalfa was sequenced under saline-alkali treatments in a previous study to explore its resistance genes (An et al., 2016). The data from the RNA-Seq showed that *MsGSTU8* was one of the genes expressed with significant difference under the saline-alkali stress conditions, suggesting that this increase in expression is a response to the environmental stress.

In this study, the *MsGSTU8* gene was isolated from alfalfa, and the expression pattern of *MsGSTU8* was characterized under different conditions. The *MsGSTU8* gene was transformed into tobacco for further analysis, and the functional characterization of the *MsGSTU8* gene was verified. This research demonstrates a potential molecular mechanism for alfalfa stress-tolerance under saline-alkali conditions.

## RESULTS

### Isolation and characterization of the *MsGSTU8* gene

**Fig. 1.**
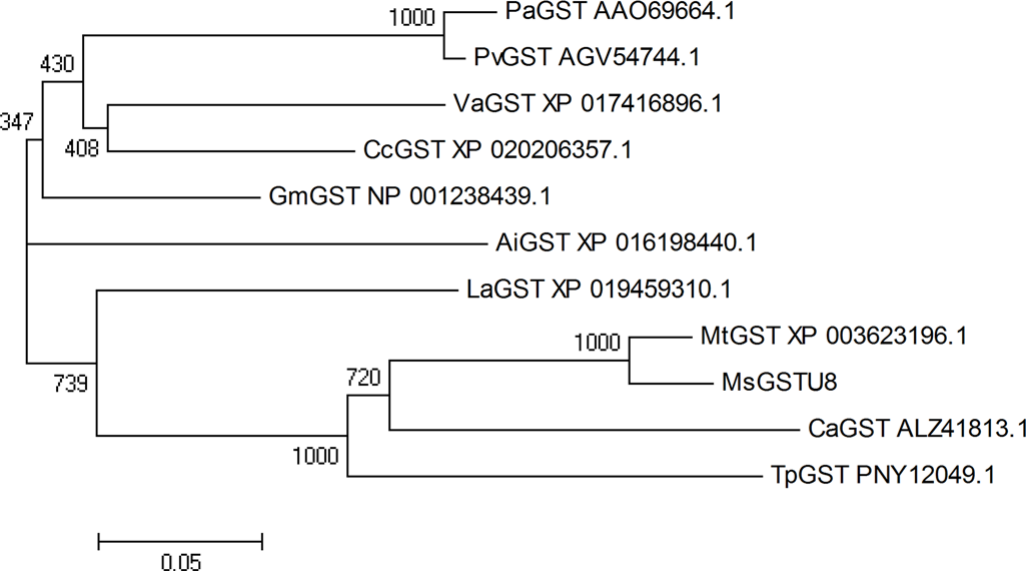
**Phylogenetic analysis of MsGSTU8 with its homologous proteins from other plant species.** The phylogenetic tree was constructed by Clustal X2 and MEGA 6.0 and performed with the Neighbor-Joining algorithm.

The *MsGSTU8* gene cloned from alfalfa had a complete open reading frame, 675 bp in length, encoding a polypeptide composed of 224 amino acid residues. The predictive analysis of MsGSTU8 showed a hydrophilic protein containing GST_N and GST_C_2 domains without transmembrane domains. The predicted molecular mass was 25.9 kDa and the isoelectric point (pI) was 5.64. The BLAST analysis indicated that the determined amino acid sequence of MsGSTU8 showed homology and high identity with the GST family members from other plant species; the highest identity (96%) was found with MtGST (XP_003623196.1). The phylogenetic tree of MsGSTU8 and its closely related members from other plant species are shown in Fig. 1.

### The MsGSTU8 protein is localized in the cytoplasm

**Fig. 2.**
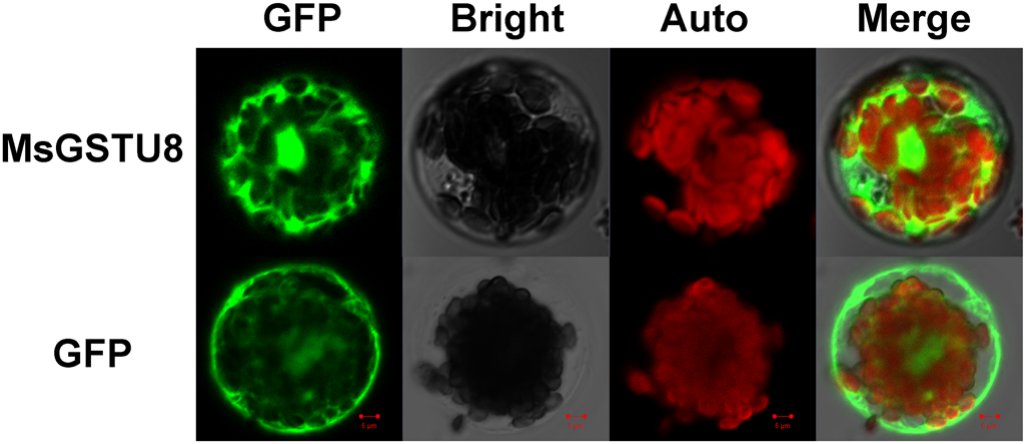
**Subcellular localization of the MsGSTU8 protein.** Transient expression of fusion protein MsGSTU8-GFP (p16318-MsGSTU8-GFP) and GFP (p16318-GFP) in *Arabidopsis* mesophyll protoplasts was analyzed by confocal laser scanning microscope. GFP, dark field; Bright, under light; Auto, red fluorescence indicates chloroplast autofluorescence; Merge, together with corresponding merged images. Scale bars: 5 µm.

The subcellular localization of MsGSTU8 was investigated using the MsGSTU8 and green fluorescence protein (GFP) fusion proteins that were overexpressed in *Arabidopsis* protoplast. The original p16318-GFP was used as a control vector. Microscopic visualization revealed that GFP fluorescence from the fusion protein was only detected in the cytoplasm of the *Arabidopsis* protoplast cells, whereas the fluorescence in the control was observed in all parts of the cell. The results suggested that MsGSTU8 is a cytoplasm-localized protein (Fig. 2).

### Expression pattern analysis of the *MsGSTU8* gene in alfalfa

**Fig. 3.**
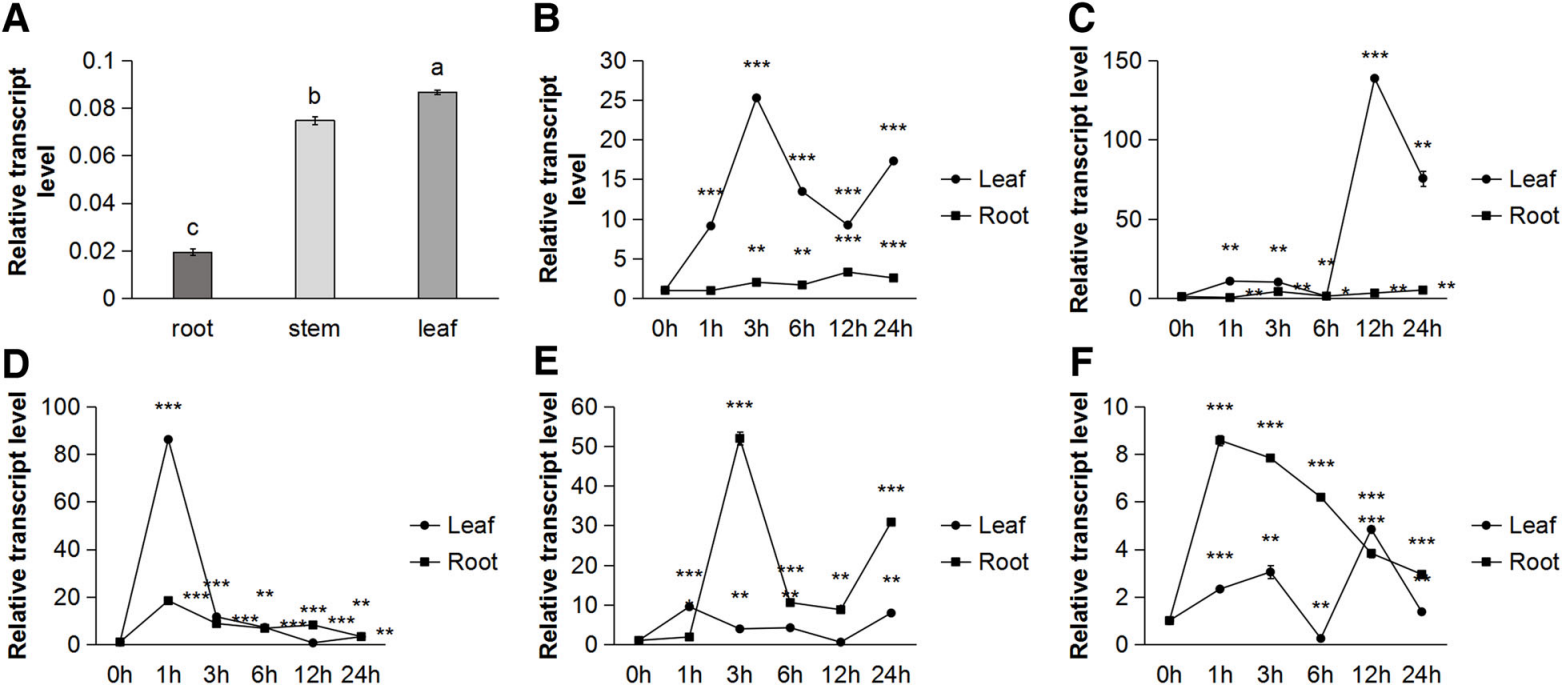
**Gene expression profile of *MsGSTU8* in alfalfa.** (A) The spatial-specific expression of *MsGSTU8* in vegetative tissues of alfalfa. (B–F) Time-course expression patterns of *MsGSTU8* in leaves and roots from alfalfa under cold treatment (B; 4°C), drought treatment (C; 400 mM mannitol), salt treatment (D; 300 mM NaCl), saline-alkali treatment (E; 100 mM Na_2_CO_3_: NaHCO_3_, 1: 2), and treatment with 100 μM ABA (F) examined by qPCR. The relative abundance of the transcripts was determined by qPCR from total RNA of the indicated organs. Values represent mean±s.d. (*n*=3). Different letters represent significant differences at (*P*<0.05) according to LSD and Duncan's multiple range tests, and asterisks indicate significant difference from control (*P<0.01, **P<0.01, ***P<0.001).

The expression pattern of *MsGSTU8* in the root, stem and leaf of alfalfa was analyzed with qPCR under normal conditions (Fig. 3A). The results showed that the *MsGSTU8* gene was expressed in different tissues and the highest expression levels were in the leaf. The *MsGSTU8* gene was induced by the cold, drought, salt, saline-alkali and abscisic acid (ABA) treatments to varying degrees in the leaf and root after exposures of 0, 1, 3, 6, 12 and 24 h (Fig. 3B–F).

### The *MsGSTU8* gene confers enhanced tolerance to saline-alkali stress in transgenic tobacco

**Fig. 4.**
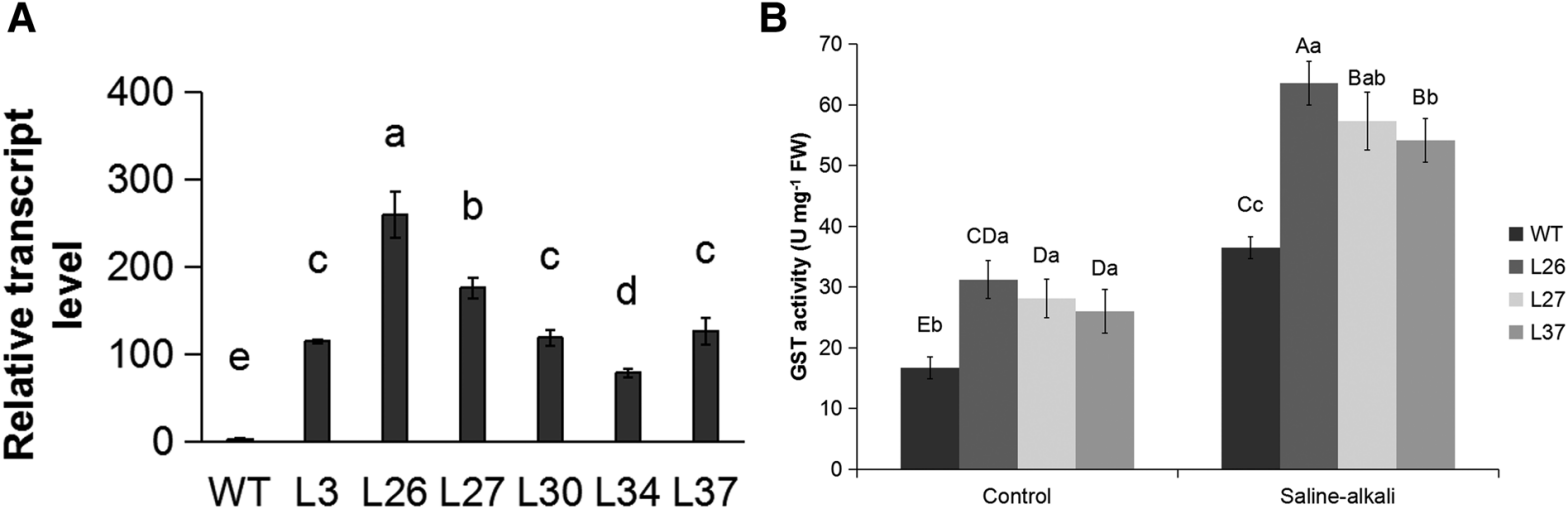
**The transcript and translation levels of *MsGSTU8* in WT and transgenic tobacco. From left to right, the four-color histogram shows WT, L26, L27 and L37.** (A) The transcript level of *MsGSTU8* in *MsGSTU8*-overexpressing tobacco assayed by qPCR. For the assay, 4-week-old tobacco leaves were harvested as samples. The mRNA level of *MsGSTU8* in WT plants were normalized as 1.0. (B) Enzymatic assay for GST using 4-week-old WT and transgenic tobacco lines under 0 mM or 30 mM NaHCO_3_. FW, fresh weight. All experiments were repeated three times. Data are the mean±s.d. of three independent experiments. Different uppercase letters and lowercase letters represent significant differences at (*P*<0.05) according to LSD and Duncan's multiple range tests.

**Fig. 5.**
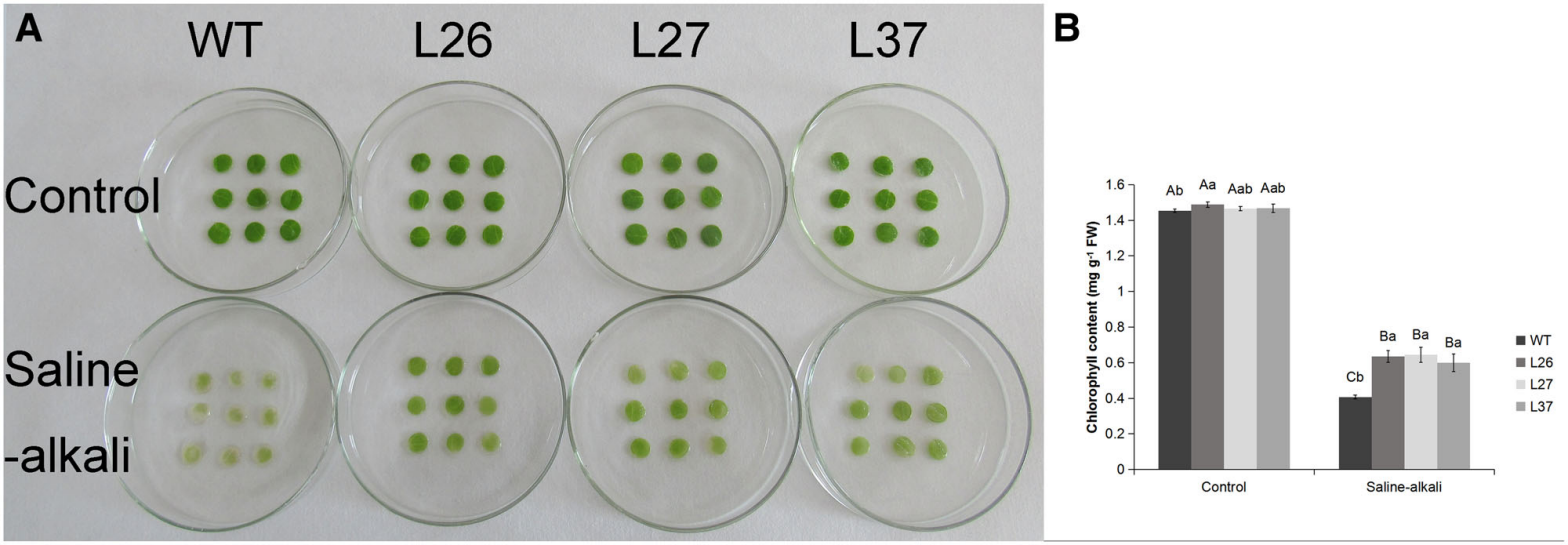
**Tolerance of transgenic tobacco to saline-alkali stress. From left to right, the four-color histogram shows WT, L26, L27 and L37.** (A) The phenotype of 4-week-old WT and transgenic tobacco line leaf discs under 30 mM NaHCO_3_ treatment for 5 days. (B) Effect of normal condition and salt-alkaline stress on chlorophyll concentration in the leaf discs of WT and transgenic tobacco lines. The data presented are the means±s.d. of three biological replicates. Different uppercase letters and lowercase letters represent significant differences at (*P*<0.05) according to LSD and Duncan’s multiple range tests.

The transgenic tobacco lines were generated by *Agrobacterium-*mediated transformations; 28 positive transgenic lines were identified from 65 transformants with glufosinate-ammonium resistance and the expression levels for the *MsGSTU8* gene were then detected. The results of the qPCR showed that the *MsGSTU8* gene was expressed in all of the transgenic lines. L26, L27 and L37 were chosen for further characterization owing to their high transcription levels of *MsGSTU8* (Fig. 4A). Leaf discs were used for the saline-alkali stress phenotype assay *in vitro*. Phenotypic observations showed that the leaf discs of wild type (WT) had greater chlorosis than the transgenic tobacco lines under 30 mM NaHCO_3_ (Fig. 5A). The relative chlorophyll content of WT was significantly lower than that of the transgenic tobacco lines (Fig. 5B) under saline-alkali conditions. This indicates that transgenic tobacco lines exhibited higher tolerance than WT to the saline-alkali treatment.

### MsGSTU8 improves the activity of GST in transgenic tobacco lines

The GST activity in the WT was significantly lower than in the transgenic tobacco lines under normal conditions. Under saline-alkali stress, both WT and transgenic tobacco had increased GST activity, but the transgenic lines had the highest activity levels (Fig. 4B).

### MsGSTU8 protein in tobacco reduces O_2_^.−^ and H_2_O_2_ levels but elevates antioxidant enzyme activities after saline-alkali stress

**Fig. 6.**
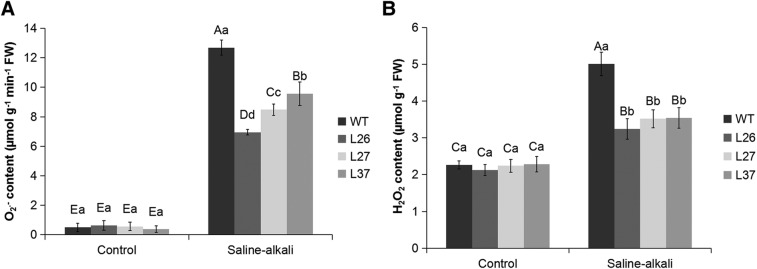
**ROS accumulation in WT and transgenic tobaccos under saline-alkali stress. From left to right, the four-color histogram shows WT, L26, L27 and L37.** Effects of normal condition and saline-alkali stress on O_2_^.−^ productivity rate (A) and H_2_O_2_ concentrations (B) in the leaves of WT and transgenic tobacco lines. Each data column represents the mean (with s.d. bar) of three replicates. One-way ANOVA post hoc multiple comparisons, LSD and Duncan's multiple range tests were selected simultaneously in equal variances assumed. The different uppercase letters and lowercase letters represent significant differences (*P*<0.05) according to LSD and Duncan's multiple range tests. The different uppercase letters represent significant differences in sample data of both control and treatment group (*P*<0.05). Different lowercase letters represent significant differences of the sample data of control or treatment group (*P*<0.05).

**Fig. 7.**
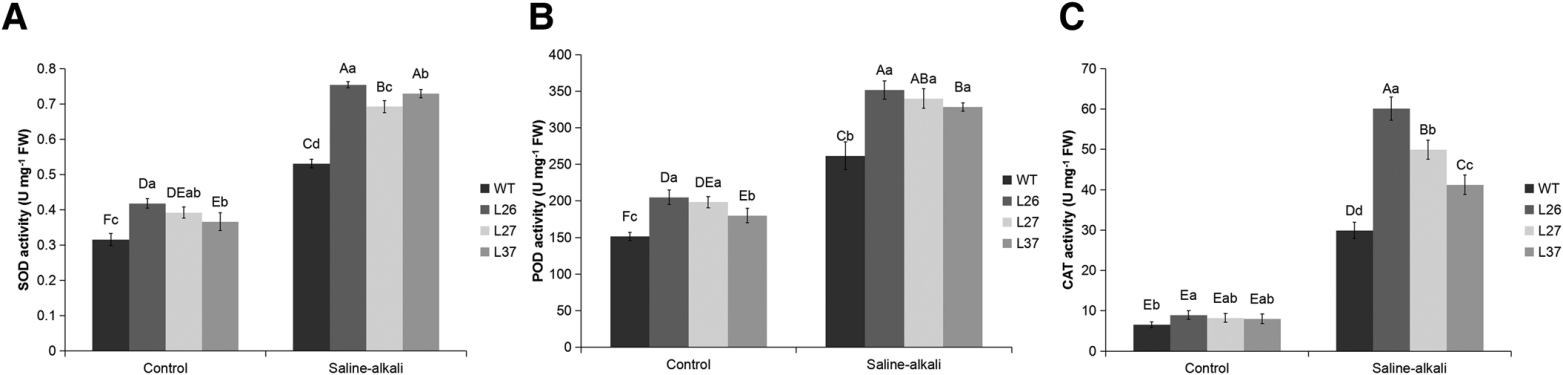
***MsGSTU8* transgenic tobacco plants show enhanced antioxidant enzyme activity under saline-alkali treatment. From left to right, the four-color histograms show WT, L26, L27 and L37.** (A–C) Variations in activity of SOD (A), POD (B) and CAT (C) in the leaves of WT and transgenic tobacco lines treated with 30 mM NaHCO_3_ in Hoagland's solution. Bars indicate standard deviations of three replicates. One-way ANOVA post hoc multiple comparisons, LSD and Duncan's multiple range tests were selected simultaneously in equal variances assumed. The different uppercase letters and lowercase letters represent significant differences (*P*<0.05) according to LSD and Duncan's multiple range tests. The different uppercase letters represent significant differences in sample data of both control and treatment group (*P*<0.05). Different lowercase letters represent significant differences of the sample data of control or treatment group (*P*<0.05).

Analysis of WT and transgenic tobacco lines showed that the accumulation of superoxide anion radical (O_2_^.−^) and hydrogen peroxide (H_2_O_2_) in the WT was more than in the transgenic tobacco lines after saline-alkali treatments (Fig. 6). The activities of superoxide dismutase (SOD), peroxidase (POD) and catalase (CAT) increased to greater extents in the transgenic tobacco than in the WT (Fig. 7).

### MsGSTU8 in transgenic tobacco decreases ion leakage and MDA levels but increases soluble sugar content under saline-alkali stress

**Fig. 8.**
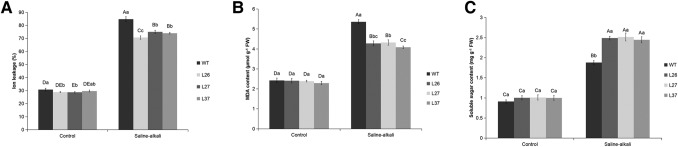
***MsGSTU8* conferred enhanced saline-alkali stress resistance by protecting the membrane from damage and maintaining osmotic pressure in transgenic tobacco. From left to right, the four-color histograms show WT, L26, L27 and L37.** (A) Effect of normal condition and saline-alkali stress on ion leakage in the leaves of WT and transgenic tobacco lines. Comparison of physiological indices between the WT and transgenic tobacco lines under saline-alkali stress. After saline-alkali treatments, the leaves of 4-week-old WT and transgenic tobacco lines were collected to measure the (B) MDA and (C) soluble sugar contents. Data are shown as the means±s.d. calculated from three biological replicates. Different uppercase letters and lowercase letters represent significant differences (*P*<0.05) according to LSD and Duncan’s multiple range tests.

To gain an insight into the degree of damage to plant cell membranes after saline-alkali treatments, the ion leakage and malondialdehyde (MDA) content were determined. The levels were higher in the WT than the transgenic tobacco lines. The accumulation of soluble sugars in the transgenic tobacco lines were higher than in the WT (Fig. 8).

### The expression pattern of stress-related genes under saline-alkali stress

**Fig. 9.**
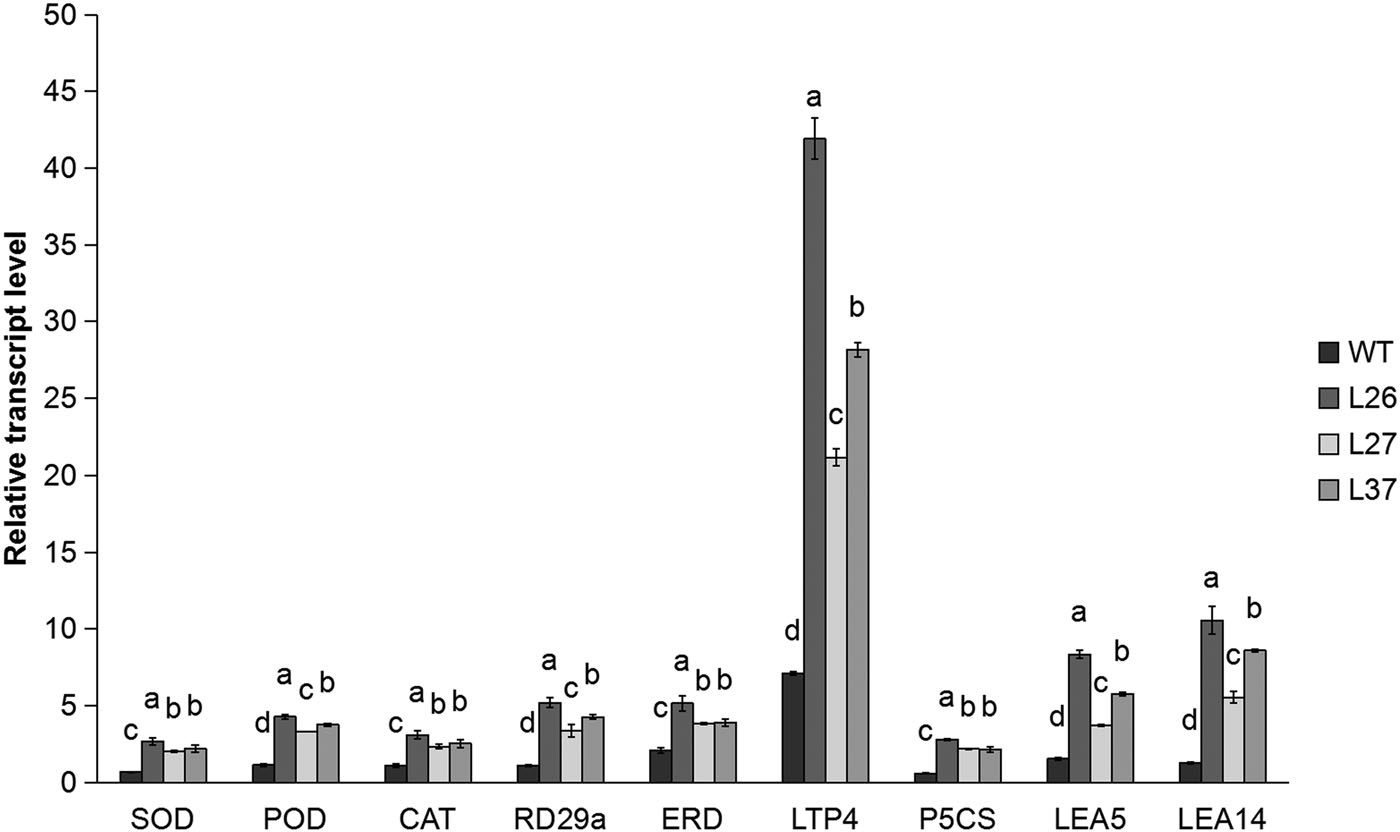
**Comparison of stress-related gene expression levels between the WT and transgenic tobacco lines after saline-alkali treatment.** The y-axis records the relative gene expression levels with *NtGAPDH* as the endogenous reference. Data are shown as the means±s.d. calculated from three biological replicates. Different letters represent significant differences at (*P*<0.05) according to LSD and Duncan's multiple range tests.

In order to shed light on the molecular events associated with *MsGSTU8* under saline-alkali stress conditions, we investigated the effect of these conditions on the expression of stress response genes. The transcript levels of three ROS detoxification genes (*NtSOD*, *NtPOD* and *NtCAT*) and six stress response genes (*NtRD29A*, *NtERD* and *NtLTP4*) and proline biosynthesis genes (*NtP5CS*, *NtLEA5* and *NtLEA14*) in the WT and transgenic tobacco lines after saline-alkali stress treatments showed that the expression of stress-related genes in transgenic tobacco lines were significantly higher in comparison to the WT after the saline-alkali treatment (Fig. 9).

## DISCUSSION

Abiotic stresses cause osmotic stress and ion toxicity that produces excessive ROS, these in turn cause oxidative stress in plants. GST is an abundant enzyme in plants encoded by an ancient and highly divergent gene superfamily with multiple functions that play active roles in the ROS scavenging pathways of plants. Previous studies have shown that overexpressing a GST gene can enhance the tolerance of transgenic plants to salt (Csiszár et al., 2014; Ji et al., 2010; Xu et al., 2015b; Zhang et al., 2018), cold (Huang et al., 2009; Roxas et al., 1997), drought (Ji et al., 2010; Lo Cicero et al., 2015; Xu et al., 2015b) and heavy metals (Dixit et al., 2011; Kumar et al., 2013); the common mechanism for the increased tolerance is reduction of oxidative damage. In previous studies, it has been widely shown that GSTs respond to salt stress and are also induced by other abiotic and biotic stresses; however, the function of GSTs in response to saline-alkali stresses were unknown. The plants were damaged by osmotic stress and ion toxicity under salt stress conditions. The same stress factors with the added influence of high-pH damage were exerted by saline-alkali stress (Yang et al., 2008). The production of several oxyradicals was caused by the high-pH stress and this changed the membrane lipid components and destroyed photosynthetic parts of the plant cells, resulting in more serious oxidative injury with saline-alkali stress than salt stress alone. The transcriptome data revealed that many of the genes in the GST family were induced by the saline-alkali stress and the *MsGSTU8* gene was significantly upregulated. The existing research indicates that the expression of certain GST genes were induced by: cold (Lo Piero et al., 2005), heavy metal ions (Shigeoka et al., 2002), salt (Diao et al., 2011), polyethylene glycol (PEG), H_2_O_2_, methylviologen (MV), ABA, salicylic acid (SA), jasmonic acid (JA), 1-naphthylacetic acid (NAA), auxin (IAA), cytokinin (CTK) and herbicides and safeners (Deridder et al., 2002) to respond to abiotic and biotic stresses (Dean et al., 2005; Gonneau et al., 2010). The transcription levels of the *MsGSTU8* gene were tested in alfalfa under cold, drought, salt and ABA treatments, and the expression of the *MsGSTU8* gene was shown to significantly increase. This shows that the *MsGSTU8* gene was induced by the stress treatments, and that the *MsGSTU8* gene might be located at the crossing point of multiple abiotic stress response pathways. The expression patterns of the *MsGSTU8* gene were analyzed in the different organs of alfalfa and were found to have their highest expression levels in the leaf. The results of subcellular localization showed that MsGSTU8 was located in the cytoplasm, indicating that MsGSTU8 probably catalyzed GSH and various toxic secondary metabolites or exogenous products produced by the plant itself in the cytoplasm, the compounds that were formed could be identified and transported across the membrane by the glutathione pump, and the conjugates were sent to the vacuoles of the plant leaves. The *MsGSTU8* gene was transformed into tobacco to determine the molecular mechanisms of plant responses to saline-alkali stress; the transcription levels of the *MsGSTU8* gene were relatively high in the transgenic tobacco lines, with L26, L27 and L37 having the highest levels. The results of the GST activity assay were consistent with the previous results of the transcription levels of the *MsGSTU8* gene, and the GST activity of the transgenic lines were found to be higher than in the WT. The phenotype showed that the leaves of WT were all whitened, while only part of the transgenic tobacco leaves showed whitening. As in earlier studies, the chlorophyll content of the plants was reduced under saline-alkali stress (Sun et al., 2014a). The chlorophyll content of transgenic tobacco lines showed a smaller decrease than that in the WT, possibly illustrating that the absorption and transduction effect of luminous energy in transgenic lines was less inhibited, relatively. For the NaHCO_3_ treatment, the high concentration of Na^+^ replaces the Ca^2+^ of the plant cytomembrane, changing the cytomembrane structure and permeability and leading to electrolyte leakage that was used as an indicator of cytomembrane damage or injury under saline-alkali stress. The greater the damage to the cytomembrane system from stress, the greater the cytomembrane permeability and the higher the relative electrical conductivity (Tantau and Dörffling, 2010). The electrolyte leakage of the transgenic tobacco line leaves increased under saline-alkali stress, but at a significantly lower level than with the WT, indicating that saline-alkali stress resulted in a change in cytomembrane permeability. The damage to the transgenic tobacco line cytomembrane system was less than that to the WT, meaning that MsGSTU8 could enhance membrane stability under saline-alkali stress, consistent with previous studies (Sun et al., 2014b). Photosynthesis was inhibited in the plant cells, and the accumulation of a large number of intracellular electrons was caused by the inhibition of the photosynthetic chain, thereby forming ROS in the chloroplasts, mitochondria and peroxisome under saline-alkali stress. Mitochondrial and chloroplast structures were degraded by excessive accumulation of ROS, affecting the structure and physiological functions of the biological macromolecules in the organelles, disrupting the normal physiological and metabolic intracellular activities (Yang et al., 2016). The ROS included the singlet oxygen (^1^O_2_), O_2_^.−^, hydroxyl radicals (HO) and H_2_O_2_ (Choudhury et al., 2017; Finkel and Holbrook, 2000; Karuppanapandian et al., 2011). Excess ROS were removed by enhancing the activity of enzymes in the antioxidant enzyme system such as SOD, POD and CAT in plants under saline-alkali stress (Bu et al., 2012). The O_2_^.−^ was proportioned into H_2_O_2_ and O_2_ by SOD which was the first line of defense against ROS; H_2_O_2_ was generated by O_2_ and H_2_O under the catalysis of POD, CAT and other antioxidant enzymes (Pandhair and Sekhon, 2006). In tomato leaves, O_2_^.−^ and H_2_O_2_ were produced by increasing the NaHCO_3_ concentration and the O_2_^.−^ and H_2_O_2_ accumulations were increased as processing time increased (Gong et al., 2013). In this study, O_2_^.−^ and H_2_O_2_ content in the transgenic tobacco lines were lower than in the WT under saline-alkali stress; this illustrates that transgenic tobacco lines were subjected to lower oxidative damage. The activity of SOD, POD and CAT in endophyte-infected rice with saline-alkali resistance was higher than in endophyte-uninfected rice which was saline-alkali sensitive under a 20 mM NaHCO_3_ treatment (Bu et al., 2012). The SOD, POD and CAT activity of transgenic tobacco lines was relatively higher than in the WT under saline-alkali stress; the high antioxidant enzyme activity of transgenic tobacco lines could clear the excess O_2_^.−^ and H_2_O_2_, maintain the balance of ROS and reduce the toxic effects of ROS by conferring tolerance to oxidative stress in the cells. The accumulation of excess ROS would cause membranous peroxidation in the cell. MDA is the end soluble product during the process of membrane lipid peroxidation, and higher content of MDA indicates more serious damage to the cell membrane system; the content of MDA is usually used as a measure of the extent of oxidative stress (Janero, 1990; Mckay and Mason, 1991). The overexpression of *GsTIFY10a* in alfalfa conferred enhanced saline-alkali tolerance with a smaller increase of MDA content in comparison with the non-transgenic alfalfa (Zhu et al., 2014). In this study, the transgenic tobacco lines had a smaller increase of MDA content compared to the WT after saline-alkali stress, indicating that MsGSTU8 can maintain an oxidation-reduction equilibrium in cells to improve tolerance to saline-alkali stress. The salt content was high in the saline-alkali soil, making water absorption through plant roots difficult due to the decline of soil osmotic potential. Plants retain water in cells by osmoregulation; micromolecule organic compounds such as soluble sugars, soluble proteins and proline are synthesized in plants. Plants resist abiotic stress by increasing the synthesis of soluble sugars; a higher content contributes to improved plant tolerance to stress (Rosa et al., 2009). In this study, the soluble sugar content of transgenic tobacco lines was significantly higher than that of the WT after saline-alkali treatment; showing that the accumulation of osmotic substances in transgenic tobacco enhanced tolerance to the saline-alkali stress. The transcriptional level of genes that encoded antioxidant enzymes and stress responsive genes were upregulated in transgenic tobacco lines under saline-alkali treatments; this indicated that the *MsGSTU8* gene overexpression in transgenic tobacco promoted the rapid response of related genes.

In conclusion, this study identifies a glutathione S-transferase family gene in alfalfa. The *MsGSTU8* gene was upregulated significantly under cold, drought, salt, saline-alkali and ABA stress treatments. The expression of the *MsGSTU8* gene was highest in the leaf of alfalfa and located in the cytoplasm. *MsGSTU8* overexpression with higher GST activity might reduce the ROS accumulation by increasing other antioxidant enzyme activities to improve osmotic regulation to relieve ROS damage; it also might influence the transcription levels of the antioxidative genes and various stress resistance-related genes in stress responsive pathways to participate in the elimination of redundant ROS in transgenic tobacco lines. The results suggest that the *MsGSTU8* gene plays an important role in saline-alkali stress responses in ABA-related regulatory systems. The *MsGSTU8* gene could be used as a potential candidate to determine the molecular mechanisms and tolerance pathways for saline-alkali stress in plants.

## MATERIALS AND METHODS

### Plant materials, growth conditions and stress treatments

Alfalfa (*Medicago sativa* L.) cultivar ‘Zhaodong’, known to have strong abiotic stress tolerance, was used in this study. The seeds were provided by the Institute of Animal Husbandry, Heilongjiang Province, China and germinated in a petri dish at room temperature for 48 h in dark conditions. After 48 h, sprouted seeds were moved into pots of vermiculite with half-strength Hoagland's solution. The seedlings were grown in an incubation room, with photoperiods of 16 h light and 8 h dark. For analysis of the *MsGSTU8* gene expression patterns, plant organs of alfalfa, including the roots, stems and leaves, were taken from 4-week-old seedlings under normal conditions. The leaves and roots of 4-week-old seedlings were used for cloning and analyzing the expression patterns of *MsGSTU8* at 0, 1, 3, 6, 12 and 24 h after cold (4°C), drought (400 mM mannitol), salt (300 mM NaCl), saline-alkali (100 mM Na_2_CO_3_: NaHCO_3_, 1: 2), and 100 μM ABA treatments.

Tobacco (*Nicotiana tabacum* ‘SR I’) seeds were surface-sterilized with sodium hypochlorite and germinated on petri dishes containing MS (Murashige and Skoog) medium (pH 5.8) solidified with 3% sucrose and 0.8% agar at 25°C. The germinating seedlings were transplanted into culture flasks with the same culture conditions. Four-week-old tobacco seedlings were used for subsequent genetic transformations.

### RNA isolation and qPCR analysis

Total RNA was extracted from the roots, stems and leaves of alfalfa for analysis of the expression pattern of *MsGSTU8*. RNAprep Pure Plant Kit (TianGen Biotech, Beijing, China) was used to extract RNA that was reverse-transcribed into cDNA by the EasyScript One-Step gDNA Removal and cDNA Synthesis SuperMix (TransGen Biotech, Beijing, China). Quantitative real-time PCR (qPCR) was performed to determine the expression pattern of *MsGSTU8* with the specific primer P1 in different organs of alfalfa under normal conditions and the leaves and roots of alfalfa were treated with cold, drought, salt, saline-alkali and ABA treatments at different time points, respectively. The *MtActin* gene was used as the endogenous control gene to normalize the expression between different samples. The qPCR was performed using an Applied Biosystems 7300 Real-Time PCR System (Applied Biosystems, Foster City, CA, USA) and quantified with the comparative 2^−ΔΔCt^ method, as previously described, to calculate the fold change of gene transcript levels. Each experiment was independently repeated in triplicate. The primers used for qPCR are listed in Table S1.

### Cloning and bioinformatics analysis of *MsGSTU8* gene

Based on the previous data of the transcriptome, the *MsGSTU8* gene was obtained from the cDNA of alfalfa under saline-alkali treatments as a template and gene-specific primer P2 (Table S1) was designed. A search of the NCBI website (https://www.ncbi.nlm.nih.gov/orffinder/) was performed with the nucleotide sequence to determine the amino acid sequence of the *MsGSTU8* gene. The SMART website (http://smart.embl-heidelberg.de/) was used to identify the domains and motifs. Predictions of the transmembrane domains were carried out on the TMHMM Server v. 2.0 website (http://www.cbs.dtu.dk/services/TMHMM/). The ExPASy website (http://web.expasy.org/protparam/) was used to predict the theoretical pI, molecular weight and hydrophilicity or hydrophobicity of the protein. The BLAST website (https://blast.ncbi.nlm.nih.gov/Blast.cgi) was used to find regions of similarity between the MsGSTU8 and other biological amino acid sequences. The related sequences were downloaded from the NCBI website to construct a phylogenetic tree using the Clustal X2 and MEGA 6.0 programs (Tamura et al., 2011; Thompson et al., 1997).

### Subcellular localization of MsGSTU8 protein assay

For GFP and *MsGSTU8* co-expressing constructions, the full-length coding sequence of the *MsGSTU8* gene without its termination codon was amplified using a suitable primer P3 (Table S1) and inserted into the *Bam*HI digested p16318-GFP vector to generate p16318-MsGSTU8-GFP. The fusion vector was introduced into *Arabidopsis* mesophyll protoplasts with p16318-GFP as the negative control. Green fluorescence from the transiently expressed constructs was observed at 488 nm using a confocal laser scanning microscope (Carl Zeiss, Jena, Germany).

### Generation of MsGSTU8 transgenic tobacco

*MsGSTU8* was ligated with a plant binary expression vector digested by the restriction sites of *Sac*I and *Pst*I to generate the recombinant constructions under the control of the cauliflower mosaic virus (CaMV) 35S promoter (Sham and Aly, 2012) with *bar* (phosphinothricin acetyltransferase) as the screening-labeled gene. The recombinant plasmid was transformed into an *Agrobacterium tumefaciens* strain EHA105 by the freeze-thaw method. The transgenic tobacco lines were obtained with *Agrobacterium*-mediated leaf disc transformation as described in a previous report (Hannah et al., 2006). Seeds of the transgenic tobacco lines were sown on MS medium containing glufosinate-ammonium (Sigma-Aldrich) to screen for the positive transgenic lines based on the resistance provided by the phosphinothricin acetyltransferase gene. To further confirm positive transgenic lines, PCR amplification of *MsGSTU8* with primer P4 (Table S1) was used, with the extracted genomic DNA as a template. The qPCR was used to investigate the transcript levels of *MsGSTU8* of the WT and transgenic tobacco lines, the *NtGAPDH* gene was used as an endogenous control (Table S1).

### Measurement of GST activity

The GST activity was assayed using Habig's method by spectrophotometry (Habig and Jakoby, 1981; Habig et al., 1974). The reactions contained 50 mM KPO_4_ (pH 6.5), 0.4 mM 1-chloro-2,4-dinitrobenzene (CDNB), 5 mM GSH, and variable aliquots of enzyme extract (25–50 μg). Reactions were initiated with the addition of the CDNB substrate in ethanol. Enzymatic formation of 2,4-dinitrophenyl-S-glutathione at 340 nm (E_340_=9.6 mM^−1^ cm^−1^) was monitored for 5 min using a spectrophotometer and corrected for non-enzymatic controls.

### Evaluation of transgenic tobacco exposed to saline-alkali stress

For saline-alkali stress tolerance assays, the leaf discs of 4-week-old WT and transgenic tobacco lines were immersed in liquid half-strength MS medium (pH 5.8) solidified with 3% sucrose containing 30 mM NaHCO_3_ for 5 days to estimate relative chlorophyll content. The chlorophylls are fat-soluble compounds that can be extracted from living plant tissue by organic solvents. The equation for 80% acetone is based on the specific absorption coefficients of Mackinney to estimate the total chlorophyll content (Lichtenthaler, 1987).

### Oxidative damage analysis

For comparing the saline-alkali stress tolerance between the tobacco lines, 4-week-old WT and transgenic tobacco seedlings at similar stages of growth were treated with half-strength Hoagland's solution containing 30 mM NaHCO_3_ for 7 days. The electrolyte/ion leakage was measured as previously described with slight modifications. Briefly, the collected leaf discs of seedlings were dipped in the deionized water at room temperature for 2 h with a vacuum pump, and the initial conductivity (C1) was measured. Then, the samples were boiled for 15 min and cooled to room temperature to measure conductivity (C2); both conductivities were measured using a conductivity meter. The relative electrolyte leakage was calculated using the formula: ion leakage=100×C1/C2 (Huang et al., 2010). The content of superoxide anion radicals (Nakajima et al., 2009) and H_2_O_2_ (Veljovic-Jovanovic et al., 2002) were measured as described in previous reports. The activity of SOD (Huang et al., 2010), CAT (Beers and Sizer, 1952) and POD (Zhao et al., 2017) were spectrophotometrically measured, which is based on the ability of antioxidant enzymes to digest corresponding substrates. The test solutions for analysis of MDA (Lutts et al., 1996) and soluble sugar content (Wei et al., 2014) were extracted from the tobacco samples, using the methodology described in previous reports.

### Stress-related gene expression analysis

The expression of antioxidative genes (*NtSOD*, *NtPOD* and *NtCAT*) and stress response genes, including those responsive to dehydration (*NtRD29A*), early response to dehydration (*NtERD*), a lipid transfer protein-coding gene (*NtLTP4*), pyrroline-5-carboxylate synthetase (*NtP5CS*) and late embryogenesis abundant protein-coding genes (*NtLEA5* and *NtLEA14*) were examined in 4-week-old WT and transgenic tobacco lines under saline-alkali stress treatments. The *NtGAPDH* gene was used as an internal control; the primers used for qPCR are listed in Table S1.

### Statistical analysis

All experiments were performed with three biological replicates with the same conditions. The data analyses were conducted with a one-sample Student's *t*-test or one-way ANOVA and shown as the mean±s.d. Statistical significance of the different methods occurred when *P*<0.05.

## Supplementary Material

Supplementary information

## References

[BIO043505C1] Abdul M. K., Nath U., Park J.-I., Biswas M. K., Choi E., Song J.-Y., Kim H.-T. and Nou I.-S. (2018). Genome-wide identification, characterization, and expression profiling of glutathione S-transferase (GST) family in pumpkin reveals likely role in cold-stress tolerance. *Genes* 9, 84 10.3390/genes9020084. PMC5852580 29439434

[BIO043505C2] An Y. M., Song L. L., Liu Y. R., Shu Y. J. and Guo C. H. (2016). De novotranscriptional analysis of alfalfa in response to saline-alkaline stress. *Front. Plant Sci.* 7, 931 10.3389/fpls.2016.00931 27458463 PMC4931813

[BIO043505C3] Axarli I., Dhavala P., Papageorgiou A. C. and Labrou N. E. (2009). Crystallographic and functional characterization of the fluorodifen-inducible glutathione transferase from Glycine max reveals an active site topography suited for diphenylether herbicides and a novel L-site. *J. Mol. Biol.* 385, 984-1002. 10.1016/j.jmb.2008.10.084 19014949

[BIO043505C4] Bechtold U. and Field B. (2018). Molecular mechanisms controlling plant growth during abiotic stress. *J. Exp. Bot.* 69, 2753-2758. 10.1093/jxb/ery157 29788471 PMC5961130

[BIO043505C5] Beers R. F. and Sizer I. W. (1952). A spectrophotometric method for measuring the breakdown of hydrogen peroxide by catalase. *J. Biol. Chem.* 195, 133-140. 14938361

[BIO043505C6] Bu N., Li X., Li Y., Ma C., Ma L. and Zhang C. (2012). Effects of Na_2_CO_3_ stress on photosynthesis and antioxidative enzymes in endophyte infected and non-infected rice. *Ecotoxicol. Environ. Saf.* 78, 35-40. 10.1016/j.ecoenv.2011.11.007 22138149

[BIO043505C7] Choudhury F. K., Rivero R. M., Blumwald E. and Mittler R. (2017). Reactive oxygen species, abiotic stress and stress combination. *Plant J.* 90, 856 10.1111/tpj.13299 27801967

[BIO043505C8] Csiszár J., Horváth E., Váry Z., Gallé Á., Bela K., Brunner S. and Tari I. (2014). Glutathione transferase supergene family in tomato: salt stress-regulated expression of representative genes from distinct GST classes in plants primed with salicylic acid. *Plant Physiol. Biochem.* 78, 15-26. 10.1016/j.plaphy.2014.02.010 24607575

[BIO043505C9] Cummins I., Cole D. J. and Edwards R. (1999). A role for glutathione transferases functioning as glutathione peroxidases in resistance to multiple herbicides in black-grass. *Plant J.* 18, 285-292. 10.1046/j.1365-313X.1999.00452.x 10377994

[BIO043505C10] Dean J. D., Goodwin P. H. and Hsiang T. (2005). Induction of glutathione S-transferase genes of Nicotiana benthamiana following infection by Colletotrichum destructivum and C. orbiculare and involvement of one in resistance. *J. Exp. Bot.* 56, 1525-1533. 10.1093/jxb/eri145 15837710

[BIO043505C11] Deridder B. P., Dixon D. P., Beussman D. J., Edwards R. and Goldsbrough P. B. (2002). Induction of glutathione S-transferases in Arabidopsis by herbicide safeners. *Plant Physiol.* 130, 1497-1505. 10.1104/pp.010066 12428014 PMC166668

[BIO043505C12] Diao G., Wang Y., Wang C. and Yang C. (2011). Cloning and functional characterization of a novel glutathione S-transferase gene from limonium bicolor. *Plant Mol. Biol. Rep.* 29, 77-87. 10.1007/s11105-010-0212-2

[BIO043505C13] Dixit P., Mukherjee P. K., Ramachandran V. and Eapen S. (2011). Glutathione transferase from Trichoderma virens enhances cadmium tolerance without enhancing its accumulation in transgenic Nicotiana tabacum. *PLoS ONE* 6, e16360 10.1371/journal.pone.0016360 21283689 PMC3024989

[BIO043505C14] Edwards R. and Dixon D. P. (2005). Plant glutathione transferases. *Methods Enzymol.* 401, 169-186. 10.1016/S0076-6879(05)01011-6 16399386

[BIO043505C15] Erpen L., Devi H. S., Grosser J. W. and Dutt M. (2018). Potential use of the DREB/ERF, MYB, NAC and WRKY transcription factors to improve abiotic and biotic stress in transgenic plants. *Plant Cell Tissue Organ Cult.* 132, 1-25. 10.1007/s11240-017-1320-6

[BIO043505C16] Finkel T. and Holbrook N. J. (2000). Oxidants, oxidative stress and the biology of ageing. *Nature* 408, 239 10.1038/35041687 11089981

[BIO043505C17] Flury T., Adam D. and Kreuz K. (1995). A 2,4-D-inducible glutathione S-transferase from soybean (Glycine max). Purification, characterisation and induction. *Physiol. Plant* 94, 312-318. 10.1034/j.1399-3054.1995.940219.x

[BIO043505C18] Gong B., Wen D., Vandenlangenberg K., Wei M., Yang F., Shi Q. and Wang X. (2013). Comparative effects of NaCl and NaHCO3 stress on photosynthetic parameters, nutrient metabolism, and the antioxidant system in tomato leaves. *Sci. Horticult.* 157, 1-12. 10.1016/j.scienta.2013.03.032

[BIO043505C19] Gonneau M., Mornet R. and Laloue M. (2010). A Nicotiana plumbaginifolia protein labeled with an azido cytokinin agonist is a glutathione S-transferase. *Physiol. Plant* 103, 114-124. 10.1034/j.1399-3054.1998.1030114.x

[BIO043505C20] Gruber M., Alahakoon U., Taheri A., Nagubushana N., Zhou R., Aung B., Sharpe A., Hannoufa A., Bonham-Smith P. and Hegedus D D. D. (2018). The biochemical composition and transcriptome of cotyledons from Brassica napus lines expressing the AtGL3 transcription factor and exhibiting reduced flea beetle feeding. *BMC Plant Biol.* 18, 64 10.1186/s12870-018-1277-6 29661140 PMC5902958

[BIO043505C21] Gu Y.-J., Han C.-L., Fan J.-W., Shi X.-P., Kong M., Shi X.-Y., Siddique K. H. M., Zhao Y.-Y. and Li F.-M. (2018). Alfalfa forage yield, soil water and P availability in response to plastic film mulch and P fertilization in a semiarid environment. *Field Crops Res.* 215, 94-103. 10.1016/j.fcr.2017.10.010

[BIO043505C22] Habig W. H. and Jakoby W. B. (1981). Assays for differentiation of glutathione S-transferases. *Methods Enzymol.* 77, 398 10.1016/S0076-6879(81)77053-8 7329316

[BIO043505C23] Habig W. H., Pabst M. J. and Jakoby W. B. (1974). Glutathione S-Transferases THE FIRST ENZYMATIC STEP IN MERCAPTURIC ACID FORMATION. *J. Biol. Chem* 249, 7130. 4436300

[BIO043505C24] Hanaka A., Lechowski L., Mroczek-Zdyrska M. and Strubińska J. (2018). Oxidative enzymes activity during abiotic and biotic stresses in Zea mays leaves and roots exposed to Cu, methyl jasmonate and Trigonotylus caelestialium. *Physiol. Mol. Biol. Plants* 24, 1-5. 10.1007/s12298-017-0479-y 29398834 PMC5787111

[BIO043505C25] Hannah M. A., Wiese D., Freund S., Fiehn O., Heyer A. G. and Hincha D. K. (2006). Natural genetic variation of freezing tolerance in arabidopsis. *Plant Physiol.* 142, 98-112. 10.1104/pp.106.081141 16844837 PMC1557609

[BIO043505C26] Horváth E., Bela K., Holinka B., Riyazuddin R., Gallé Á., Hajnal Á., Hurton Á., Fehér A. and Csiszár J. (2019). The Arabidopsis glutathione transferases, AtGSTF8 and AtGSTU19 are involved in the maintenance of root redox homeostasis affecting meristem size and salt stress sensitivity. *Plant Sci.* 283, 366-374. 10.1016/j.plantsci.2019.02.005 31128707

[BIO043505C27] Huang C., Guo T., Zheng S. C., Feng Q. L., Liang J. H. and Li L. (2009). Increased cold tolerance in Arabidopsis thaliana transformed with Choristoneura fumiferana glutathione S-transferase gene. *Biol. Plantarum* 53, 183-187. 10.1007/s10535-009-0031-1

[BIO043505C28] Huang X.-S., Liu J.-H. and Chen X.-J. (2010). Overexpression of PtrABF gene, a bZIP transcription factor isolated from Poncirus trifoliata, enhances dehydration and drought tolerance in tobacco via scavenging ROS and modulating expression of stress-responsive genes. *BMC Plant Biol.* 10, 230 10.1186/1471-2229-10-230 20973995 PMC3017851

[BIO043505C29] Janero D. R. (1990). Malondialdehyde and thiobarbituric acid-reactivity as diagnostic indices of lipid peroxidation and peroxidative tissue injury. *Free Radic. Biol. Med.* 9, 515-540. 10.1016/0891-5849(90)90131-2 2079232

[BIO043505C30] Ji W., Zhu Y., Li Y., Yang L., Zhao X., Cai H. and Bai X. (2010). Over-expression of a glutathione S-transferase gene, GsGST, from wild soybean (Glycine soja) enhances drought and salt tolerance in transgenic tobacco. *Biotechnol. Lett.* 32, 1173 10.1007/s10529-010-0269-x 20383560

[BIO043505C31] Karuppanapandian T., Juncheol M., Changsoo K., Manoharan K. and Wook K. (2011). Reactive oxygen species in plants: their generation, signal transduction, and scavenging mechanisms. *Aust. J. Crop Sci.* 5, 709-725.

[BIO043505C32] Klay I., Gouia S., Liu M., Mila I., Khoudi H., Bernadac A., Bouzayen M. and Pirrello J. (2018). Ethylene Response Factors (ERF) are differentially regulated by different abiotic stress types in tomato plants. *Plant Sci.* 274, 137-145. 10.1016/j.plantsci.2018.05.023 30080597

[BIO043505C33] Kong L., Deng H., Hu S., Wang F., Miao L., Chen C., Zhao K. and Yu X. (2018). Isolation, expression, and evolution analysis of the type 2C protein phosphatase gene BcABI1 involved in abiotic and biotic stress in Brassica campestris ssp. chinensis. *Plant Growth Regul.* 85, 317-327. 10.1007/s10725-018-0399-z

[BIO043505C34] Kumar S. and Trivedi P. K. (2018). Glutathione S-transferases: role in combating abiotic stresses including arsenic detoxification in plants. *Front. Plant Sci.* 9, 751 10.3389/fpls.2018.00751 29930563 PMC5999759

[BIO043505C35] Kumar S., Asif M. H., Chakrabarty D., Tripathi R. D., Dubey R. S. and Trivedi P. K. (2013). Expression of a rice Lambda class of glutathione S-transferase, OsGSTL2, in Arabidopsis provides tolerance to heavy metal and other abiotic stresses. *J. Hazard. Mater.* 248-249, 228-237. 10.1016/j.jhazmat.2013.01.004 23380449

[BIO043505C36] Lichtenthaler H. K. (1987). [34] Chlorophylls and carotenoids: pigments of photosynthetic biomembranes. *Methods Enzymol.* 148, 350-382. 10.1016/0076-6879(87)48036-1

[BIO043505C37] Liu C. Y., Song Y. Y., Ren H. N., Sun G. G., Liu R. D., Jiang P., Long S. R., Zhang X., Wang Z. Q. and Cui J. (2017). Cloning and expression of a Trichinella spiralis putative glutathione S-transferase and its elicited protective immunity against challenge infections. *Parasit. Vectors* 10, 448 10.1186/s13071-017-2384-1 28962639 PMC5622431

[BIO043505C38] Lo Cicero L., Madesis P., Tsaftaris A. and Lo Piero A. R. (2015). Tobacco plants over-expressing the sweet orange tau glutathione transferases (CsGSTUs) acquire tolerance to the diphenyl ether herbicide fluorodifen and to salt and drought stresses. *Phytochemistry* 116, 69-77. 10.1016/j.phytochem.2015.03.004 25819876

[BIO043505C39] Lo Piero A. R., Puglisi I., Rapisarda P. and Petrone G. (2005). Anthocyanins accumulation and related gene expression in red orange fruit induced by low temperature storage. *J. Agric. Food Chem.* 53, 9083-9088. 10.1021/jf051609s 16277406

[BIO043505C40] Lutts S., Kinet J. M. and Bouharmont J. (1996). NaCl-induced senescence in leaves of rice (Oryza sativa L.) cultivars differing in salinity resistance. *Ann. Bot.* 78, 389-398. 10.1006/anbo.1996.0134

[BIO043505C41] Manuka R., Saddhe A. A. and Kumar K. (2018). Expression of OsWNK9 in Arabidopsis conferred tolerance to salt and drought stress. *Plant Sci.* 270, 58-71. 10.1016/j.plantsci.2018.02.008 29576087

[BIO043505C43] Mckay H. M. and Mason W. L. (1991). Physiological indicators of tolerance to cold storage in Sitka spruce and Douglas-fir seedlings. *Can. J. Forest Res.* 21, 890-901. 10.1139/x91-124

[BIO043505C44] Moons A. (2003). Osgstu3 and osgtu4, encoding tau class glutathione S-transferases, are heavy metal- and hypoxic stress-induced and differentially salt stress-responsive in rice roots. *FEBS Lett.* 553, 427-432. 10.1016/S0014-5793(03)01077-9 14572664

[BIO043505C45] Moons A. (2005). Regulatory and functional interactions of plant growth regulators and plant glutathione S-Transferases (GSTs). *Vitamins Hormones* 72, 155-202. 10.1016/S0083-6729(05)72005-7 16492471

[BIO043505C46] Moreno J. E., Moreno-Piovano G. and Chan R. L. (2018). The antagonistic basic helix-loop-helix partners BEE and IBH1 contribute to control plant tolerance to abiotic stress. *Plant Sci.* 271, 143 10.1016/j.plantsci.2018.03.024 29650152

[BIO043505C47] Nakajima Y., Tsuruma K., Shimazawa M., Mishima S. and Hara H. (2009). Comparison of bee products based on assays of antioxidant capacities. *BMC Complement. Altern. Med.* 9, 4 10.1186/1472-6882-9-4 19243635 PMC2664783

[BIO043505C48] Pandhair V. and Sekhon B. S. (2006). Reactive oxygen species and antioxidants in plants: an overview. *J. Plant Biochem. Biotechnol.* 15, 71-78. 10.1007/BF03321907

[BIO043505C49] Qu A.-L., Ding Y.-F., Jiang Q. and Zhu C. (2013). Molecular mechanisms of the plant heat stress response. *Biochem. Biophys. Res. Commun.* 432, 203 10.1016/j.bbrc.2013.01.104 23395681

[BIO043505C50] Rosa M., Prado C., Podazza G., Interdonato R., González J. A., Hilal M. and Prado F. E. (2009). Soluble sugars: metabolism, sensing and abiotic stress: a complex network in the life of plants. *Plant Signal. Behav.* 4, 388-393. 10.4161/psb.4.5.8294 19816104 PMC2676748

[BIO043505C51] Roxas V. P. Jr., Smith R. K., Allen E. R. and Allen R. D. (1997). Overexpression of glutathione S-transferase/glutathione peroxidase enhances the growth of transgenic tobacco seedlings during stress. *Nat. Biotechnol.* 15, 988 10.1038/nbt1097-988 9335051

[BIO043505C52] Sandermann H. Jr. (1992). Plant metabolism of xenobiotics. *Trends Biochem. Sci.* 17, 82-84. 10.1016/0968-0004(92)90507-6 1566333

[BIO043505C53] Seppänen M. M., Alitalo V., Bäckström H. K., Mäkiniemi K., Jokela V., Falghera-Winseman L. and Khazaei H. (2018). Growth, freezing tolerance, and yield performance of alfalfa (Medicago sativa L.) cultivars grown under controlled and field conditions in northern latitudes. *Canadian Journal of Plant Science* 98, 1109-1118. 10.1139/cjps-2017-0305

[BIO043505C54] Sham A. and Aly M. A. M. (2012). Bioinformatics based comparative analysis of omega-3 fatty acids in desert plants and their role in stress resistance and tolerance. *Int. J. Plant Res.* 2, 80-89. 10.5923/j.plant.20120203.06

[BIO043505C55] Shameer S. and Prasad T. N. V. K. V. (2018). Plant growth promoting rhizobacteria for sustainable agricultural practices with special reference to biotic and abiotic stresses. *Plant Growth Regul.* 84, 603-615. 10.1007/s10725-017-0365-1

[BIO043505C56] Shigeoka S., Ishikawa T., Tamoi M., Miyagawa Y., Takeda T., Yabuta Y. and Yoshimura K. (2002). Regulation and function of ascorbate peroxidase isoenzymes. *J. Exp. Bot.* 53, 1305-1319. 10.1093/jexbot/53.372.1305 11997377

[BIO043505C57] Singh A., Giri J., Kapoor S., Tyagi A. K. and Pandey G. K. (2010). Protein phosphatase complement in rice: genome-wide identification and transcriptional analysis under abiotic stress conditions and reproductive development. *BMC Genomics* 11, 435-435. 10.1186/1471-2164-11-435 20637108 PMC3091634

[BIO043505C58] Sun M., Sun X., Zhao Y., Zhao C., Duanmu H., Yu Y., Ji W. and Zhu Y. (2014a). Ectopic expression of GsPPCK3 and SCMRP in Medicago sativa enhances plant alkaline stress tolerance and methionine content. *PLoS ONE* 9, e89578 10.1371/journal.pone.0089578 24586886 PMC3934933

[BIO043505C59] Sun X., Yang S., Sun M., Wang S., Ding X., Zhu D., Ji W., Cai H., Zhao C. and Wang X. (2014b). A novel Glycine soja cysteine proteinase inhibitor GsCPI14, interacting with the calcium/calmodulin-binding receptor-like kinase GsCBRLK, regulated plant tolerance to alkali stress. *Plant Mol. Biol.* 85, 33-48. 10.1007/s11103-013-0167-4 24407891

[BIO043505C60] Suzuki N., Miller G., Sejima H., Harper J. and Mittler R. (2013). Enhanced seed production under prolonged heat stress conditions in Arabidopsis thaliana plants deficient in cytosolic ascorbate peroxidase 2. *J. Exp. Bot.* 64, 253 10.1093/jxb/ers335 23183257 PMC3528037

[BIO043505C61] Tamura K., Peterson D., Peterson N., Stecher G., Nei M. and Kumar S. (2011). MEGA5: molecular evolutionary genetics analysis using maximum likelihood, evolutionary distance, and maximum parsimony methods. *Mol. Biol. Evol.* 28, 2731 10.1093/molbev/msr121 21546353 PMC3203626

[BIO043505C62] Tantau H. and Dörffling K. (2010). In vitro-selection of hydroxyproline-resistant cell lines of wheat (Triticum aestivum): accumulation of proline, decrease in osmotic potential, and increase in frost tolerance. *Physiol. Plant* 82, 243-248. 10.1111/j.1399-3054.1991.tb00088.x

[BIO043505C63] Thompson J. D., Gibson T. J., Plewniak F., Jeanmougin F. and Higgins D. G. (1997). The CLUSTAL_X windows interface: flexible strategies for multiple sequence alignment aided by quality analysis tools. *Nucleic Acids Res.* 25, 4876-4882. 10.1093/nar/25.24.4876 9396791 PMC147148

[BIO043505C64] Traewachiwiphak S., Yokthongwattana C., Ves-Urai P., Charoensawan V. and Yokthongwattana K. (2018). Gene expression and promoter characterization of heat-shock protein 90B gene (HSP90B) in the model unicellular green alga Chlamydomonas reinhardtii. *Plant Sci.* 272, 107-116. 10.1016/j.plantsci.2018.04.010 29807581

[BIO043505C65] Veljovic-Jovanovic S., Noctor G. and Foyer C. H. (2002). Are leaf hydrogen peroxide concentrations commonly overestimated? The potential influence of artefactual interference by tissue phenolics and ascorbate. *Plant Physiol. Biochem.* 40, 501-507. 10.1016/S0981-9428(02)01417-1

[BIO043505C66] Wang Z., Huang S., Jia C., Liu J., Zhang J., Xu B. and Jin Z. (2013). Molecular cloning and expression of five glutathione S-transferase (GST) genes from Banana (Musa acuminata L. AAA group, cv. Cavendish). *Plant Cell Rep.* 32, 1373-1380. 10.1007/s00299-013-1449-7 23652818

[BIO043505C67] Wei J., Cui-Yun W. U., Jiang Y. and Wang H. L. (2014). Sample preparation optimization for determination of soluble sugar in red jujube fruits by anthrone method. *Food Sci.* 37, 168-176.

[BIO043505C68] Xu J., Tian Y.-S., Xing X.-J., Peng R.-H., Zhu B., Gao J.-J. and Yao Q.-H. (2015a). Over-expression of AtGSTU19 provides tolerance to salt, drought and methyl viologen stresses in Arabidopsis. *Physiol. Plant* 156, 164-175. 10.1111/ppl.12347 25975461

[BIO043505C70] Yang C., Shi D. and Wang D. (2008). Comparative effects of salt and alkali stresses on growth, osmotic adjustment and ionic balance of an alkali-resistant halophyte Suaeda glauca (Bge.). *Plant Growth Regul.* 56, 179 10.1007/s10725-008-9299-y

[BIO043505C71] Yang G., Xu Z., Peng S., Sun Y., Jia C. and Zhai M. (2016). In planta characterization of a tau class glutathione S-transferase gene from Juglans regia (JrGSTTau1) involved in chilling tolerance. *Plant Cell Rep.* 35, 681 10.1007/s00299-015-1912-8 26687965

[BIO043505C72] Yang Q., Liu Y.-J. and Zeng Q.-Y. (2019). Overexpression of three orthologous glutathione S-transferases from Populus increased salt and drought resistance in Arabidopsis. *Biochem. Syst. Ecol.* 83, 57-61. 10.1016/j.bse.2019.01.001

[BIO043505C73] Ye Y., Ding Y., Jiang Q., Wang F., Sun J. and Zhu C. (2017). The role of receptor-like protein kinases (RLKs) in abiotic stress response in plants. *Plant Cell Rep.* 36, 235-242. 10.1007/s00299-016-2084-x 27933379

[BIO043505C74] Yu C., Song L., Song J., Ouyang B., Guo L., Shang L., Wang T., Li H., Zhang J. and Ye Z. (2018). ShCIGT, a Trihelix family gene, mediates cold and drought tolerance by interacting with SnRK1 in tomato. *Plant Sci.* 270, 140-149. 10.1016/j.plantsci.2018.02.012 29576067

[BIO043505C75] Zhang Y., Zhao H., Zhou S., He Y., Luo Q., Zhang F., Qiu D., Feng J., Wei Q., Chen L. et al. (2018). Expression of TaGF14b, a 14-3-3 adaptor protein gene from wheat, enhances drought and salt tolerance in transgenic tobacco. *Planta* 248, 117 10.1007/s00425-018-2887-9 29616395

[BIO043505C76] Zhao X., Wei P., Liu Z., Yu B. and Shi H. (2017). Soybean Na+/H+ antiporter GmsSOS1 enhances antioxidant enzyme activity and reduces Na+ accumulation in Arabidopsis and yeast cells under salt stress. *Acta Physiol. Plant.* 39, 19 10.1007/s11738-016-2323-3

[BIO043505C77] Zhao P., Wang D., Wang R., Kong N., Zhang C., Yang C., Wu W., Ma H. and Chen Q. (2018). Genome-wide analysis of the potato Hsp20 gene family: identification, genomic organization and expression profiles in response to heat stress. *BMC Genomics* 19, 61 10.1186/s12864-018-4443-1 29347912 PMC5774091

[BIO043505C78] Zhou Y.-B., Liu C., Tang D.-Y., Yan L., Wang D., Yang Y.-Z., Gui J.-S., Zhao X.-Y., Li L.-G., Tang X.-D. et al. (2018). The receptor-like cytoplasmic kinase STRK1 phosphorylates and activates CatC, thereby regulating H2O2 homeostasis and improving salt tolerance in rice. *Plant Cell* 30, 1100-1118. 10.1105/tpc.17.01000 29581216 PMC6002193

[BIO043505C79] Zhu D., Li R., Liu X., Sun M., Wu J., Zhang N. and Zhu Y. (2014). The positive regulatory roles of the TIFY10 proteins in plant responses to alkaline stress. *PLoS ONE* 9, e111984 10.1371/journal.pone.0111984 25375909 PMC4222965

