# Supplementary material for: Overexpression of an alfalfa glutathione S-transferase gene improved the saline-alkali tolerance of transgenic tobacco
Source: Biol Open. 2019 Aug 30;8(9):bio043505. doi: 10.1242/bio.043505 (PMC6777358; doi:10.1242/bio.043505)
Supplement: Supplementary information [file biolopen-8-043505-s1.pdf]

Table S1 List of primers used for PCR or qPCR analysis

Primer name	Primer sequence	Comments
P1	F: TAGAAAAGGGAGGGAACT<br>R: CGTGCAGCACTTAAACAC	Primer sequences were used for qPCR assay
P2	F: CCTTGGCTCGTTTCTGGACC<br>R: CGGTACCCGGGGATCCTCTA	Primer sequences were used in *MsGSTU8* cloning
P3	F: TATCTCTAGAGGATCCATGGCATCAAATCAG<br>R: TGCTCACCATGGATCCTTTTGAAGCAAGAAGACT	Primer sequences were used to construct vector for subcellular localization assay
P4	F: CCTTGGCTCGTTTCTGGACC<br>R: CGGTACCCGGGGATCCTCTA	Primer sequences were used to identify transgenic tobaccos
MtActin	F: ACGAGCGTTTCAGATG<br>R: ACCTCCGATCCAGACA	Primer sequences were used for qPCR assay
NtGAPDH	F: TAAGGGTGGTGCCAAGAAGGT<br>R: AGCAAGAGGAGCAAGGCAGTT	Primer sequences were used for qPCR assay
NtSOD	F: TGCGTACAAAGCAAATAGAC<br>R: ATTGATATGTAGTCAGGGCG	Primer sequences were used for qPCR assay
NtPOD	F: GATCTTTTTGTTGAACCTGG<br>R: TAATTTGTCTGAACTTCCGC	Primer sequences were used for qPCR assay
NtCAT	F: ATTTCTTCTCTTTCCTTCCG<br>R: TATGATTTGTACCTCCGACC	Primer sequences were used for qPCR assay
NtRD29a	F: TCGGTGTACCAACAGGCATA<br>R: CCCTTGCTTTGGTGTTGTTT	Primer sequences were used for qPCR assay
NtERD	F: ACGTGGAGGCTACAGATCGTGGTTTG<br>R: TCTCCACTGGTACAGCCGTGTCCTCAC	Primer sequences were used for qPCR assay
NtLTP4	F: ATGCTGCAGTGGGATTAAGG<br>R: AGCAGTCAATGGAAGGGCTA	Primer sequences were used for qPCR assay
NtP5CS	F: TTCCAGACGTCTTCAGGCAC<br>R: TCATATCCGGCCTGTTGAGC	Primer sequences were used for qPCR assay
NtLEA5	F: TGTTAGCAGGCGTGGGTAT<br>R: CTCTCGCTCTTGTTGGGTTC	Primer sequences were used for qPCR assay
NtLEA14	F: CTCCGTTCCCGTACCTATCA<br>R: CAATCTGCGCCAATATCCTT	Primer sequences were used for qPCR assay

Biology Open • Supplementary information
